# A Simple Wide Range Approximation of Symmetric Binomial Distribution

**DOI:** 10.3390/e27010021

**Published:** 2024-12-30

**Authors:** Tamás Szabados

**Affiliations:** Department of Stochastics, Institute of Mathematics, Budapest University of Technology and Economics, Muegyetem rkp. 3, H ep, 5 em, 1521 Budapest, Hungary; szabados@math.bme.hu; Tel.: +36-30-390-0660

**Keywords:** wide range, approximation, binomial distribution, 60E15, 60F05

## Abstract

The paper gives a wide range, uniform, local approximation of symmetric binomial distribution. The result clearly shows how one has to modify the classical de Moivre–Laplace normal approximation in order to give an estimate at the tail as well as to minimize the relative error.

The topic of this paper is a wide range, uniform, local approximation of symmetric binomial distribution, an extension of the classical de Moivre–Laplace theorem in the symmetric case [1] (Ch. VII) [2]. In other words, I would like to approximate individual binomial probabilities not only in a classical neighborhood of the center, but at the tail as well. The result clearly shows how one has to modify the normal approximation in order to give a wide range estimate to minimize the relative error. The method will be somewhat similar to the ones applied by [3,4,5] or in the proof of Tusnády’s lemma, see, e.g., [6]; however, my task is much simpler than those. This simplification makes the proof short, transparent and natural. Moreover, the result is non-asymptotic, that is, it gives explicit, nearly optimal, upper and lower bounds for the relative error with a finite *n*. Thus, I hope that it can be used in both applications and teaching.

Let ${\left(X_{r}\right)}_{r\geq 1}$ be a sequence of independent, identically distributed random steps with $P\left(X_{r}=\pm 1\right)=\frac{1}{2}$ and $S_{\ell }=\sum_{r=1}^{\ell }X_{r}$(ℓ≥1), $S_{0}=0$, be the corresponding simple, symmetric random walk. Then

(1) P(Sℓ=j)=ℓℓ+j22−ℓ(|j|≤ℓ).

Here, we use the convention that the above binomial coefficient is zero whenever ℓ+j is not divisible by 2. Since $P\left(S_{\ell }=j\right)=P\left(S_{\ell }=-j\right)$ for any *j*, it is enough to consider only the case j≥0 whenever it is convenient.

First we consider the case when ℓ=2n, even. Let us introduce the notation

ak,n:=P(S2n=2k)=2nn+k2−2n(|k|≤n).

Also, introduce the notation

(2) $$b_{k,n}:=n\left\{\left(1+\frac{k+\frac{1}{2}}{n}\right)\log\left(1+\frac{k}{n}\right)+\left(1-\frac{k-\frac{1}{2}}{n}\right)\log\left(1-\frac{k}{n}\right)\right\}$$

when n≥1 and |k|<n, and

(3) $$b_{\pm n,n}:=\left(2n+\frac{1}{2}\right)\log2-\frac{1}{2}\log\left(2\pi n\right)\;\left(n\geq 1\right).$$

**Theorem** **1.** *(a) For any n≥1 and |k|≤n, we have*

(4) $$a_{k,n}\leq \frac{1}{\sqrt{\pi n}}e^{-b_{k,n}}.$$

*(b) For any n≥1 and |k|≤rn, r∈(0,1), we have*

(5) $$a_{k,n}>\frac{1}{\sqrt{\pi n}}e^{-b_{k,n}}\;\exp\left(-\frac{1}{7n}-\frac{r^{4}}{3{\left(1-r^{2}\right)}^{2}\;n}\right).$$

*(c) In accordance with the classical de Moivre–Laplace normal approximation, for n≥3 and $\left|k\right|\leq n^{\frac{2}{3}}$ one has*

(6) $$1-2n^{-\frac{1}{3}}<\frac{a_{k,n}}{\frac{1}{\sqrt{\pi n}}e^{-\frac{k^{2}}{n}}}<1+2n^{-\frac{1}{3}}.$$

**Proof.**  As usual, the first step is to estimate the central term

a0,n=2nn2−2n=(2n)!(n!)22−2n.

By Stirling’s formula, see, e.g., [1] (p. 54), we have:

(7) $$\sqrt{2\pi n}{\left(\frac{n}{e}\right)}^{n}e^{\frac{1}{12n+1}}<n!<\sqrt{2\pi n}{\left(\frac{n}{e}\right)}^{n}e^{\frac{1}{12n}}\;\left(n\geq 1\right).$$

Thus, after simplification we obtain

(8) $$\frac{1}{\sqrt{\pi n}}e^{-\frac{1}{7n}}<a_{0,n}<\frac{1}{\sqrt{\pi n}}e^{-\frac{1}{9n}}\;\left(n\geq 1\right).$$

Second, also by the standard way, for 1≤k≤n,

$$a_{k,n}=a_{0,n}\frac{n(n-1)\cdots (n-k+1)}{\left(n+1)(n+2)\cdots (n+k\right)}=a_{0,n}\frac{\left(1-\frac{1}{n}\right)\left(1-\frac{2}{n}\right)\cdots \left(1-\frac{k-1}{n}\right)}{\left(1+\frac{1}{n}\right)\left(1+\frac{2}{n}\right)\cdots \left(1+\frac{k}{n}\right)}.$$

So it follows that

(9) $$\begin{array}{c} \log a_{k,n}=\log a_{0,n}-\log\left(1+\frac{k}{n}\right)-2\sum_{j=1}^{k-1}\frac{1}{2}\log\frac{1+\frac{j}{n}}{1-\frac{j}{n}}\\ =\log a_{0,n}-\log\left(1+\frac{k}{n}\right)-2\sum_{j=1}^{k-1}\tanh^{-1}\left(\frac{j}{n}\right). \end{array}$$

To approximate the sum here, let us introduce the integral

(10) $$I\left(x\right):=\int_{0}^{x}\tanh^{-1}\left(t\right)\;dt=\frac{1}{2}\left(1+x\right)\log\left(1+x\right)+\frac{1}{2}\left(1-x\right)\log\left(1-x\right)$$

for |x|<1, and its approximation by a trapezoidal sum

(11) $$T_{k,n}:=\frac{1}{n}\left\{\frac{1}{2}\tanh^{-1}\left(0\right)+\sum_{j=1}^{k-1}\tanh^{-1}\left(\frac{j}{n}\right)+\frac{1}{2}\tanh^{-1}\left(\frac{k}{n}\right)\right\},$$

where 0≤k<n. It is well-known that the error of the trapezoidal formula for a function $f\in C^{2}\left(\left[a,b\right]\right)$ is

$$T_{n}\left(f\right)-\int_{a}^{b}f\left(t\right)\;dt=\frac{{\left(b-a\right)}^{3}}{12n^{2}}f^{\prime \prime }\left(x\right),\;x\in \left[a,b\right].$$

Since ${\left(\tanh^{-1}\right)}^{\prime \prime }\left(x\right)=2x{\left(1-x^{2}\right)}^{-2}$, we obtain that

(12) $$0\leq T_{k,n}-I\left(\frac{k}{n}\right)\leq \frac{r^{4}}{6{\left(1-r^{2}\right)}^{2}\;n^{2}},\;\mathrm{when}\;0\leq k\leq rn,\;r\in \left(0,1\right).$$

Let us combine Formulas (8)–(12):

$$\log a_{k,n}=\log a_{0,n}-2nT_{k,n}-\frac{1}{2}\log\left(1+\frac{k}{n}\right)-\frac{1}{2}\log\left(1-\frac{k}{n}\right),$$

thus

(13) $$\log\frac{1}{\sqrt{\pi n}}-b_{k,n}-\frac{1}{7n}-\frac{r^{4}}{3{\left(1-r^{2}\right)}^{2}\;n}<\log a_{k,n}<\log\frac{1}{\sqrt{\pi n}}-b_{k,n}-\frac{1}{9n},$$

where 0≤k≤rn and

(14) $$b_{k,n}=2nI\left(\frac{k}{n}\right)+\frac{1}{2}\log\left(1-\frac{k^{2}}{n^{2}}\right).$$

Clearly, (14) is the same as (2). Thus, (13) proves (a) and (b) of the theorem.

Let us see now, using Taylor expansions, a series expansion of $b_{k,n}$ when |k|<n. First, for |x|<1,

$$I\left(x\right)=\frac{x^{2}}{1\cdot 2}+\frac{x^{4}}{3\cdot 4}+\frac{x^{6}}{5\cdot 6}+\frac{x^{8}}{7\cdot 8}+\cdots .$$

Second, also for |x|<1,

$$\log\left(1-x^{2}\right)=-x^{2}-\frac{x^{4}}{2}-\frac{x^{6}}{3}-\frac{x^{8}}{4}-\cdots .$$

So by (14), for |k|<n we have a convergent series for $b_{k,n}$:

(15) $$\begin{array}{cc} b_{k,n} & =\frac{k^{2}}{n}\left(1-\frac{1}{2n}\right)+\frac{k^{4}}{2n^{3}}\left(\frac{1}{3}-\frac{1}{2n}\right)+\frac{k^{6}}{3n^{5}}\left(\frac{1}{5}-\frac{1}{2n}\right)+\cdots \\ & =\sum_{j=1}^{\infty }{\left(\frac{k}{n}\right)}^{2j}\frac{1}{j}\left(\frac{n}{2j-1}-\frac{1}{2}\right). \end{array}$$

By (15), for n≥3 and $\left|k\right|\leq n^{\frac{2}{3}}$ we obtain that

$$\begin{array}{c} \left|b_{k,n}-\frac{k^{2}}{n}\right|\leq \frac{n^{-\frac{2}{3}}}{2}+\sum_{j=2}^{\infty }n^{-\frac{2}{3}j}\frac{1}{j}\left(\frac{n}{2j-1}+\frac{1}{2}\right)\\ \leq \frac{n^{-\frac{2}{3}}}{2}+\frac{n}{2}\left(\frac{1}{3}+\frac{1}{6}\right)\sum_{j=2}^{\infty }n^{-\frac{2}{3}j}\leq n^{-\frac{1}{3}}. \end{array}$$

Thus, by (4), for n≥3 and $\left|k\right|\leq n^{\frac{2}{3}}$,

(16) $$a_{k,n}\leq \frac{1}{\sqrt{\pi n}}e^{-\frac{k^{2}}{n}}\;e^{n^{-1/3}}<\frac{1}{\sqrt{\pi n}}e^{-\frac{k^{2}}{n}}\;\left(1+2n^{-\frac{1}{3}}\right).$$

Similarly, by (5), for n≥3 and $\left|k\right|\leq n^{\frac{2}{3}}$,

(17) $$\begin{array}{c} a_{k,n}>\frac{1}{\sqrt{\pi n}}e^{-\frac{k^{2}}{n}}\;\exp\left(-\frac{1}{7n}-\frac{n^{-\frac{7}{3}}}{3{\left(1-n^{-\frac{2}{3}}\right)}^{2}}-n^{-\frac{1}{3}}\right)\\ \geq \frac{1}{\sqrt{\pi n}}e^{-\frac{k^{2}}{n}}\;e^{-1.21n^{-1/3}}>\frac{1}{\sqrt{\pi n}}e^{-\frac{k^{2}}{n}}\;\left(1-2n^{-1/3}\right). \end{array}$$

(16) and (17) together prove (c) of the theorem. □

The main moral of Theorem 1 is that the exponent $k^{2}/n$ in the exponent of the normal approximation is only a first approximation of the series (15). In (c), the bound $\left|k\right|\leq n^{\frac{2}{3}}$ was chosen somewhat arbitrarily. It is clear from the series (15) that the bound should be $o(n^{\frac{3}{4}})$. The above given bound was picked because it seemed to be satisfactory for usual applications with large deviation and gave a nice relative error bound $2n^{-\frac{1}{3}}$.

It is not difficult to extend the previous results to the odd-valued case of the symmetric binomial probabilities

ak,n*:=P(S2n−1=2k−1)=2n−1n+k−12−2n+1(−n+1≤k≤n).

Define

(18) $$b_{k,n}^{*}:=n\left\{\left(1+\frac{k-\frac{1}{2}}{n}\right)\log\left(1+\frac{k}{n}\right)+\left(1-\frac{k-\frac{1}{2}}{n}\right)\log\left(1-\frac{k}{n}\right)\right\}$$

for n≥1 and |k|<n, and

(19) $$b_{n,n}^{*}:=\left(2n-\frac{1}{2}\right)\log2-\frac{1}{2}\log\left(2\pi n\right)\;\left(n\geq 1\right).$$

**Theorem** **2.** *(a) For any n≥1 and −n+1≤k≤n, we have*

(20) $$a_{k,n}^{*}<\frac{1}{\sqrt{\pi n}}e^{-b_{k,n}^{*}}\;\exp\left(\frac{2}{3n}\right).$$

*(b) For any n≥1 and |k|≤rn, r∈(0,1), we have*

(21) $$a_{k,n}^{*}>\frac{1}{\sqrt{\pi n}}e^{-b_{k,n}^{*}}\;\exp\left(-\frac{1}{n}-\frac{r^{4}}{3{\left(1-r^{2}\right)}^{2}\;n}\right).$$

*(c) In accordance with the classical de Moivre–Laplace normal approximation, for n≥4 and $\left|k\right|\leq n^{\frac{2}{3}}$ one has*

(22) $$1-3n^{-\frac{1}{3}}<\frac{a_{k,n}^{*}}{\frac{1}{\sqrt{\pi n}}e^{-\frac{k^{2}}{n}}}<1+6n^{-\frac{1}{3}}.$$

**Proof.**  Since this proof is very similar to the previous one, several details are omitted. First, by Stirling’s Formula (7), after simplifications we obtain that

(23) $$\frac{1}{\sqrt{\pi n}}e^{-\frac{1}{n}}<a_{0,n}^{*}<\frac{1}{\sqrt{\pi n}}e^{\frac{2}{3n}}\;\left(n\geq 1\right).$$

Second, similarly to (13), for n≥1 and |k|≤rn, r∈(0,1), we obtain that

(24) $$\log\frac{1}{\sqrt{\pi n}}-b_{k,n}^{*}-\frac{1}{n}-\frac{r^{4}}{3{\left(1-r^{2}\right)}^{2}\;n}<\log a_{k,n}^{*}<\log\frac{1}{\sqrt{\pi n}}-b_{k,n}^{*}+\frac{2}{3n},$$

where

(25) $$b_{k,n}^{*}:=2nI\left(\frac{k}{n}\right)-\tanh^{-1}\left(\frac{k}{n}\right)=\sum_{j=1}^{\infty }{\left(\frac{k}{n}\right)}^{2j-1}\frac{1}{2j-1}\left(\frac{k}{j}-1\right).$$

(25) clearly agrees with (18). (24) proves (a) and (b).

By the series in (25), for n≥4 and $\left|k\right|\leq n^{\frac{2}{3}}$ we obtain that

$$\left|b_{k,n}^{*}-\frac{k^{2}}{n}\right|<2n^{-\frac{1}{3}}.$$

This and (24) imply (c). □

## Data Availability

No new data were created or analyzed in this study. Data sharing is not applicable to this article.
